# Scalable Production of Recombinant Adeno-Associated Virus Vectors Expressing Soluble Viral Receptors for Broad-Spectrum Inhibition of Porcine Reproductive and Respiratory Syndrome Virus Type 2

**DOI:** 10.3390/vetsci12040366

**Published:** 2025-04-14

**Authors:** Xiaoming Liu, Nuo Xu, Xiaoli Song, Linlin Zhuang, Qiuping Shen, Huaichang Sun

**Affiliations:** 1 The Department of Animal Husbandry and Veterinary Medicine, Jiangsu Vocational College of Agriculture and Forestry, Jurong 212400, China; nuoxu@jsafc.edu.cn (N.X.); zhuanglinlin@jsafc.edu.cn (L.Z.); shenqiuping@jsafc.edu.cn (Q.S.); 2 The College of Veterinary Medicine, Yangzhou University, Yangzhou 225009, China; 3 Jiangsu Provincial Animal Disease Control Center, 124 Caochangmen Street, Nanjing 210036, China; sxl_gadpcc@126.com

**Keywords:** PRRSV, soluble viral receptor fusions, adeno-associated virus vector, insect cell bioreactor, broad-spectrum antiviral activity

## Abstract

Porcine reproductive and respiratory syndrome virus (PRRSV) continues to be a major threat to the global swine industry, causing significant economic losses. To address this, we developed a scalable recombinant adeno-associated virus (rAAV)-based strategy for the delivery of soluble viral receptors (SVRs) to treat and potentially eliminate PRRSV infections. This strategy involves fusing the virus-binding domains of two key cellular receptors, sialoadhesin (Sn4D) and CD163 (SRCR5-9), with an Fc fragment. We then used an insect cell–baculovirus expression vector system to produce the rAAV-SRCR59-Fc/Sn4D-Fc vector. Through a series of optimizations, we determined the best conditions for rAAV production, including a baculovirus co-infection ratio of 0.5:1.0, an initial insect cell density of 2.0 × 10^6^ cells/mL, a fetal bovine serum concentration of 2%, and a culture temperature of 30 °C. Under these optimized conditions, we achieved a high titer of rAAV-SRCR59-Fc/Sn4D-Fc in a 2 L bioreactor, reaching 5.4 ± 0.9 × 10^9^ infectious viral particles (IVPs)/mL. Notably, in vitro neutralization assays using a Transwell co-culture system demonstrated a 4.3 log reduction in viral titers across genetically diverse PRRSV-2 strains, including VR2332, JXA1, JS07, and SH1705. Collectively, this study provides a robust platform for large-scale rAAV production and highlights the potential of SVR-based gene therapy to address the antigenic diversity of PRRSV-2.

## Simple Summary

We developed a scalable insect cell–baculovirus system to produce rAAV vectors expressing dual soluble viral receptors (Sn4D-Fc/SRCR59-Fc) against PRRSV. By systematically optimizing baculovirus co-infection ratio (0.5:1.0), initial cell density (2.0 × 10^6^ cells/mL), fetal bovine serum concentration (2%), and temperature (30 °C), we achieved a scalable production of rAAV-SRCR59-Fc/Sn4D-Fc vectors with a titer of 5.4 ± 0.9 × 10^9^ infectious viral particles (IVPs)/mL in a 2 L bioreactor, representing a 170-fold increase compared with conventional flask-based methods. In vitro tests showed these SVRs reduced viral titers of diverse PRRSV strains by ~4.3 log, demonstrating broad-spectrum antiviral activity. This platform offers a durable, scalable solution to combat PRRSV, addressing vaccine limitations for swine health management.

## 1. Introduction

Porcine reproductive and respiratory syndrome (PRRS) is a highly contagious swine infectious disease caused by porcine reproductive and respiratory syndrome virus (PRRSV), which is characterized by late-term maternal reproductive failure, increased mortality of newborn piglets, and respiratory disease in growing pigs [1,2]. PRRSV is classified into two major genotypes: European (Type 1) and North American (Type 2), which exhibit ~60% nucleotide identity [3,4]. Currently, the North American genotype remains the dominant circulating strain in China. At present, PRRS is mainly controlled by immunization with attenuated live vaccines [5]. Although the vaccine could induce relative immune protection against homologous strain infection, the immune protection against heterologous strains is debatable due to the risk of virulence conversion and virion spread [6,7]. In addition, PRRSV is a highly variable RNA virus with complex infection and immune evasion mechanisms [8,9]. Therefore, the improvement of new PRRS vaccines faces serious challenges, and a new anti-PRRSV strategy is urgently needed.

Porcine alveolar macrophages (PAMs) serve as the primary target cells for PRRSV-2 infection, with sialoadhesin (Sn) and CD163 acting as critical entry receptors [10,11]. Sn, a PAM-restricted immunoglobulin superfamily receptor containing 17 Ig-like domains, mediates viral attachment via its N-terminal Sn4D domains in a sialic acid-dependent manner, interacting specifically with the M/GP5 glycoprotein complex of PRRSV-2 [12]. CD163, a PAM-specific scavenger receptor cysteine-rich (SRCR) superfamily protein, has been identified as the cellular receptor for PRRSV-2 glycoprotein 2/4 (GP2/GP4). This interaction occurs through its fifth SRCR domain to facilitate viral entry, while domains 6–9 (SRCR59) are essential for maintaining structural integrity and enabling efficient virus-receptor interaction [11]. These findings highlight the potential of soluble viral-binding domains Sn4D and SRCR5-9 as novel antiviral agents. Previous studies using recombinant adenovirus vectors demonstrated that these soluble viral receptors (SVRs) effectively blocked PRRSV-2 infection both in vitro and in vivo; however, their therapeutic utility was limited by short-term transgene expression [13,14]. To address this, we recently developed a recombinant adeno-associated virus (rAAV) vector expressing dual SVRs (Sn4D-SRCR59-Fc and SRCR59-Fc/Sn4D-Fc) fused with porcine IgG1 Fc, achieving prolonged transgene expression in murine models [15].

rAAV is generally considered a promising gene transfer vector with low immunogenicity and long-term expression in animal models or clinical therapy [16,17]. Some investigators have demonstrated that some rAAV serotypes show high efficiency for gene transfer in specific tissues [18,19]. However, the classic low-titer production mode of rAAV cannot meet the requirements for large animal studies or human clinical trials. The insect cell–baculovirus expression vector system has been discovered as a reproducible, facile, and adaptable process to produce rAAV, with high biological interest [20,21]. In this study, we developed an optimized insect cell–baculovirus system to achieve scalable production of rAAV-SRCR59-Fc/Sn4D-Fc vectors, yielding high titers of 5.4  ±  0.9  ×  10^9^ infectious viral particles (IVPs)/mL in a 2 L bioreactor. In vitro assays demonstrated that these vectors potently blocked infection by diverse PRRSV-2 strains (VR2332, JXA1, JS07, and SH1705) with a ~4.3 log reduction in viral titers, highlighting their broad-spectrum antiviral potential.

## 2. Materials and Methods

### 2.1. Cells

*Spodoptera frugiperda* (Sf9) cells (Invitrogen, Carlsbad, CA, USA) were seeded at 27 °C in ExpiSf™ CD medium (Gibco, Billings, MT, USA) augmented with 1% penicillin/streptomycin. The monkey kidney cell line Marc-145 (ATCC, Manassas, VA, USA) and the human embryonic kidney cell line HEK-293A (Agilent Technologies, Santa Clara, CA, USA) were seeded in Dulbecco’s modified Eagle medium (DMEM, Gibco) containing 10% fetal bovine serum (FBS). Primary PAM cells were isolated from Landrace pigs [22], and the SV40-transformed PAM cell line 3D4/21 (ATCC, Manassas, VA, USA) was cultured in RPMI 1640 medium supplemented with 10% FBS, 2 mM L-glutamine, 1% non-essential amino acids, and 1 mM sodium pyruvate (Invitrogen, Carlsbad, CA, USA).

### 2.2. Virus

Recombinant Bac-RC (encoding AAV-2 rep and cap genes) and recombinant Bac-SRCR59-Fc/Sn4D-Fc (encoding the SRCR59-Fc/Sn4D-Fc transgene) were propagated and titrated in Sf9 cells [15]. PRRSV strain VR2332 (ATCC VR2332) is a prototype strain of genotype 2 [13]. PRRSV strains JXA1 [23], JS07 [24], and SH1705 [14] are highly pathogenic strains isolated in China. The four PRRSV-2 strains were propagated and titrated on MARC-145 cells.

### 2.3. Optimization of the Conditions for rAAV Production

rAAV-SRCR59-Fc/Sn4D-Fc was generated via co-infection of Sf9 cells with two recombinant baculoviruses: rBac-SRCR59-Fc/Sn4D-Fc and rBac-RC. Prior to optimization, rBac-RC and rBac-SRCR59-Fc/Sn4D-Fc were titrated on Sf9 cells by real-time fluorescence quantitative PCR (RTFQ PCR) using TB Green^®^ Premix ExTaq™ II Kit (Invitrogen) and the primer pairs listed in Table 1. For optimization, Sf9 cells were cultured in 125 mL shake flasks (CORNING, Corning, NY, USA) with 20 mL medium. Initially, cells (1.0 × 10^6^ cells/mL) were co-infected with rBac-SRCR59-Fc/Sn4D-Fc at different multiplicities of infection (MOI: 0.1, 0.5, 1.0, 5.0, or 10) and rBac-RC (MOI 1.0) to evaluate the effect of baculovirus concentration ratios on rAAV production. Subsequent experiments varied the initial insect cell density (0.5, 1.0, 2.0, or 3.0 × 10^6^ cells/mL), fetal bovine serum concentration (0%, 2%, 5%, 10%), and incubation temperature (24 °C, 27 °C, 30 °C, or 33 °C). Viral titers were quantified using a standardized AAV-293 cell-based immunofluorescence assay with FITC-conjugated goat anti-pig IgG (KPL, Gaithersburg, MD, USA) at a 1:200 dilution, followed by automated image analysis (ImageXpress Micro XLS, Molecular Devices, San Jose, CA, USA) to count fluorescence-forming units (FFUs). Statistical significance was determined via two-tailed Student’s *t*-test or Kruskal–Wallis test using SPSS 22.0.

### 2.4. Preparation of Baculovirus-Infected Insect Cells (BIICs)

Under the optimized conditions, Sf9 cells were grown in 125 mL shake flasks with 20 mL of medium and co-infected with rBac-SRCR59-Fc/Sn4D-Fc and rBac-RC. When the cell diameter became larger and the cell viability remained >90%, the baculovirus-infected insect cells (BIICs) were recovered by low-speed centrifugation (300× *g*, for 10 min) and resuspended in serum-free cell cryopreservation culture medium (NCM Biotech, Suzhou, China). One-milliliter aliquots containing 1.0 × 10⁷ cells were dispensed into cryotubes (CORNING, Corning, NY, USA) and placed at a temperature of −80 °C.

### 2.5. Large-Scale rAAV Production

The two-liter bioreactor (BioFlo 320, Eppendorf, Hamburg, Germany) was filled with the minimum working volume of insect serum-free growth medium. Sf9 cells (7.5 × 10^5^/mL) were inoculated into the rocking platform bioreactor after the temperature was maintained at 27 °C, and 30% O_2_ in an air mixture was provided as previously described [25]. Detect the cell number in a timely manner and perform fed-batch with fresh 2% FBS medium to maintain a cell density of 1.6~1.8 × 10^6^/mL [26]. Once the final working volume and a cell density of 2.0 × 10^6^ cells/mL were reached, thawed BIICs aliquots (BIICs:Sf9 cells = 10⁻^3^) were added to the culture with the temperature adjusted to 30 °C. Cell density or viability was measured using trypan blue dye exclusion and an automated cell counter (Countess™ 3 FL, Invitrogen, Carlsbad, CA, USA), and the same volume of cell medium was collected and rAAV was titrated every 24 h until t = 216 h post-infection (hpi). After the cell diameter became larger and the proportion of infected cells exceeded 90%, the cells were centrifuged for 15 min at 2500× *g* and suspended in lysis buffer (50 mM HEPES, 2 mM MgCl_2_, pH 7.5) for rAAV purification.

### 2.6. Purification of Recombinant Adeno-Associated Viruses

After three cycles of freeze–thawing in lysis buffer, the cells were treated with benzonase nuclease (50 U/mL) and MgSO_4_ (37.5 mM) and disrupted two times at 1200 bar using a High-Pressure Cell Disruptor (JNBIO, Guangzhou, China). The supernatants were extracted with 10% chloroform following centrifugation for 15 min at 4 °C at 10,000× *g* as previously described [15]. Next, the upper aqueous phases were incubated with 25 mM CaCl_2_ for 1 h on ice. After 15 min of centrifugation at 10,000× *g* at 4 °C, the supernatants were incubated with 0.62 M NaCl and 8% PEG8000 for 3 h on ice as previously described [27]. Following 30 min of centrifugation at 3000× *g* at 4 °C, the purified rAAV was suspended in PBS buffer and titrated on AAV-293 cells.

### 2.7. Transmission Electron Microscopy

The purified rAAV was absorbed onto copper grids (400-mesh) at room temperature for 2.5 min and dried with filter paper. The grids were stained with 3% phosphotungstic acid for 2.5 min and observed under a transmission electron microscope (Tecnai 12, Philips, Eindhoven, Netherlands) at an acceleration voltage of 75 kV.

### 2.8. Immunofluorescence

AAV-293 or 3D4/21 cells were seeded onto 6-well plates and transduced individually with the rAAV-SRCR59-Fc/Sn4D-Fc. At 48 h post-transduction (HPT), the cells were fixed with 4% paraformaldehyde, permeabilized with 0.5% Triton X-100, and blocked in PBSM (5% defatted milk powder in PBS, pH 7.4). Immunofluorescence was performed as previously described [28] using anti-pig IgG.

### 2.9. Western Blotting

The purified rAAV was separated by 12% SDS–PAGE and transferred onto a nitrocellulose membrane (Merk, Darmstadt, Germany) using a Trans-Blot Turbo instrument (Bio-Rad, Hercules, CA, USA). After blocking with 5% skim milk powder in PBST (0.1% Tween 20 in PBS), Western blotting was performed using homemade mouse anti-AAV serum (1:200) as the first antibody and DyLight 800-labelled goat anti-mouse IgG (1:10,000; KPL, Gaithersburg, MD, USA) as the second antibody. The hybridization signals were scanned using an Infrared Imaging System (Odyssey DLX, LI–COR, Lincoln, NE, USA) at 800 nm as previously described [15]. The SVR fusions SRCR59-Fc and Sn4D-Fc were purified using Protein A Sefinose Columns (Sangon, Shanghai, China) following the manufacturer’s instructions, as previously described [13]. The purified proteins (200 ng) were separated by 12% SDS–PAGE and analyzed by Western blotting using DyLight 800-labelled goat anti-pig IgG (1:10,000), as previously described [15].

### 2.10. Viral Infection Blocking Assay

To evaluate the anti-PRRSV activity of the two SVR fusion proteins (SRCR59-Fc and Sn4D-Fc) against diverse PRRSV strains (VR2332, JXA1, JS07, and SH1705), 3D4/21 cells were cultured in the upper chambers of 24-well Transwell plates (CORNING, Corning, NY, USA) and transduced with rAAV-SRCR59-Fc/Sn4D-Fc at a fixed MOI of 100, with empty AAV serving as the negative control. At 48 h post-transduction, PAM cells in the lower chambers were infected with the indicated PRRSV strains at an MOI of 0.5. After 24 h of co-cultivation, PAM cells were lysed by three cycles of freeze–thawing (−80 °C for 10 min/37 °C for 5 min) to release viral particles for PRRSV titration on MARC-145 cells using the 50% tissue culture infective dose (TCID50) assay as previously described [29]. All experiments were performed in triplicate, and data were analyzed using Student’s *t*-test.

### 2.11. Statistical Analysis

Statistical analysis was performed using SPSS Statistics 22. The results were considered to be statistically significant at *p* < 0.05 or extremely significant at *p* < 0.01. Three independent assays were performed for each separate data point, and the results are presented as the mean ± standard deviation (SD).

## 3. Results

### 3.1. Determination of the Optimal Packaging Conditions for rAAV

Considering that an excessively high viral titer can induce rapid cell death and subsequently compromise the packaging efficiency of rAAV, this study primarily investigated the influence of the baculovirus co-infection ratio on AAV production. Sf9 cells were seeded at an initial density of 1.0 × 10^6^ cells/mL into 125 mL shake flasks filled with 20 mL of medium supplemented with 10% FBS. The cultures were maintained at 27 °C with a shaking speed of 100 rpm. Subsequently, Sf9 cells were co-infected with rBac-SRCR59-Fc/Sn4D-Fc at different multiplicities of infection (MOIs: 0.1, 0.5, 1.0, 5.0, or 10) and rBac-RC at an MOI of 1.0. After 72 h of co-infection, rAAVs were collected and titrated on AAV-293 cells by immunofluorescence using FITC-labeled goat anti-pig IgG and expressed as FFUs/mL. The results demonstrated that when the co-infection was carried out at an MOI ratio of 0.5:1.0 (rBac-SRCR59-Fc/Sn4D-Fc:rBac-RC), the rAAV achieved the highest titer of 4.0 ± 0.5 × 10^6^ IVPs/mL. This titer was highly significantly greater than those at MOI ratios of 5.0:1.0 (1.7 ± 0.4 × 10^6^ IVPs/mL) and 10:1.0 (0.6 ± 0.2 × 10^6^ IVPs/mL). Moreover, it was significantly higher than those at 1.0:1.0 (2.4 ± 0.1 × 10^6^ IVPs/mL) and 0.1:1.0 (2.6 ± 0.1 × 10^6^ IVPs/mL).

**Figure 1:**
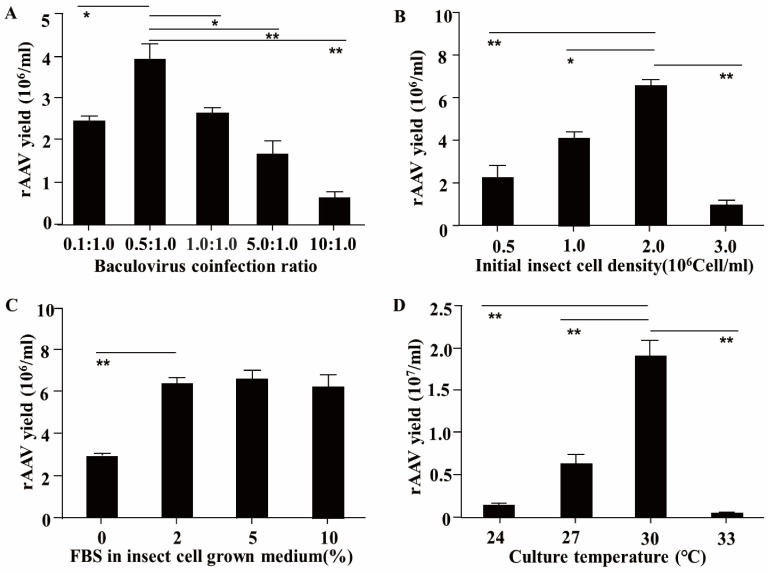
Optimization of rAAV production parameters. (**A**) Effect of baculovirus co-infection ratio (rBac-SRCR59-Fc/Sn4D-Fc:rBac-RC) on rAAV packaging efficiency. Sf9 cells (1.0 × 10^6^ cells/mL) were co-infected at MOI ratios of 0.1:1.0, 0.5:1.0, 1.0:1.0, 5.0:1.0, or 10:1.0. The highest rAAV titer (4.0 ± 0.5 × 10^6^ IVPs/mL) was achieved at an MOI ratio of 0.5:1.0, which was significantly higher than other ratios (*p* < 0.01 for 5.0:1.0 and 10:1.0; *p* < 0.05 for 1.0:1.0 and 0.1:1.0). (**B**) Impact of initial insect cell density on rAAV yield. Maximum production (6.1 ± 0.1 × 10^6^ IVPs/mL) occurred at 2.0 × 10^6^ cells/mL, showing significant improvement compared to 0.5 × 10^6^ (*p* < 0.01), 3.0 × 10^6^ (*p* < 0.01), and 1.0 × 10^6^ (*p* < 0.05) cells/mL. (**C**) Influence of fetal bovine serum (FBS) concentration on rAAV production. Optimal yields were observed in 2% FBS medium (6.1 ± 0.1 × 10^6^ IVPs/mL), which was significantly higher than serum-free conditions (*p* < 0.01) but showed no significant difference from 5% or 10% FBS groups. (**D**) Effect of culture temperature on rAAV titers. The highest titer (1.9 ± 0.3 × 10^7^ IVPs/mL) was achieved at 30 °C, significantly surpassing 27 °C (*p* < 0.01) and 24 °C (*p* < 0.01). No infectious virus was detected at 33 °C. No infectious virus was detected at 33 °C. ** indicates a highly significant difference (*p* < 0.01) and * indicates a significant difference (*p* < 0.05) between experimental groups (two-tailed Student’s *t*-test). Data are presented as mean ± SD from three independent biological replicates. Error bars represent SD.

Testing initial insect cell densities demonstrated that the rAAV packaging yield was maximized at densities of 2.0 × 10^6^ cells/mL (6.1 ± 0.1 × 10^6^ IVPs/mL), which was highly significantly higher than yields observed at densities of 0.5 × 10^6^ cells/mL (0.5 ± 0.1 × 10^6^ IVPs/mL) or 3.0 × 10^6^ cells/mL (2.1 ± 0.3 × 10^6^ IVPs/mL), and significantly higher than densities of 1.0 × 10^6^ cells/mL (4.0 ± 0.1 × 10^6^ IVPs/mL) (Figure 1B). The concentration of FBS in insect cell growth medium also affected the production of rAAV. The highest rAAV packaging yields was observed in insect media containing 2% (6.1 ± 0.1 × 10^6^ IVPs/mL), 5% (6.2 ± 0.3 × 10^6^ IVPs/mL), or 10% (6.0 ± 0.3 × 10^6^ IVPs/mL) FBS, compared to serum-free medium (2.5 ± 0.1 × 10^6^ IVPs/mL) (Figure 1C). In addition, when considering the culture temperature, the number of rAAVs at 30 °C (1.9 ± 0.3 × 10^7^ IVPs/mL) was highly significantly higher than that at 27 °C (6.1 ± 0.1 × 10^6^ IVPs/mL) or 24 °C (1.0 ± 0.1 × 10^6^ IVPs/mL), and no viral infectivity was detected at 33 °C (Figure 1D).

In conclusion, based on the experimental findings, the optimal conditions for rAAV production are as follows: co-infect Sf9 cells with rBac-SRCR59-Fc/Sn4D-Fc and rBac-RC at a MOI ratio of 0.5:1.0, an initial insect cell density of 2.0 × 10^6^ cells/mL, 2% FBS in the insect cell growth medium, and a culture temperature of 30 °C. These optimized conditions will be applied to the subsequent large-scale production of rAAV.

### 3.2. Baculovirus-Infected Insect Cells (BIICs) Production

**Figure 2:**
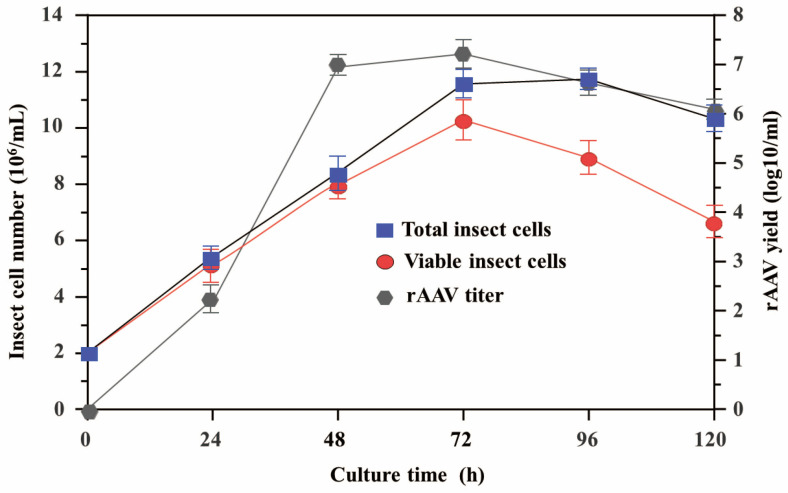
Time course of baculovirus co-infection in the cell flask. To determine the optimum harvest time of BIICs under the optimized baculovirus co-infection conditions, the insect cell density, the number of living cells, and the yields of rAAV were checked at different times after infection.

Sf9 cells were cultured in 125 mL shake flasks with 20 mL medium (2 × 10^6^ cells/mL) supplemented with 2% FBS. The cells were co-infected with rBac-SRCR59-Fc/Sn4D-Fc (MOI 0.5) and rBac-RC (MOI 1.0) at a temperature of 30 °C. Insect cell density, viability, and rAAV yields were measured at 24, 48, 72, 96, and 120 h post-infection (hpi). As revealed in Figure 2, the highest rAAV titer was observed at 72 hpi (1.9 ± 0.6 × 10^7^ IVPs/mL), followed by 48 hpi (1.5 ± 0.5× 10^7^ IVPs/mL), 96 hpi (5.2 ± 0.4× 10^6^ IVPs/mL), and 120 hpi (0.8 ± 0.6 × 10^6^ IVPs/mL). Only small amounts of infectious particles were detected at 24 hpi (2.6 ± 0.5 × 10^2^ IVPs/mL). The insect cell viability at 72 hpi, 96 hpi, or 120 hpi was under 90%, while the cell viability remained >90% at 24 or 48 h post-infection (Figure 2). Although the highest rAAV titer was at 72 hpi, considering factors such as maintaining relatively high cell viability and facilitating subsequent operations, 48 hpi was identified as the optimal time for BIICs collection.

### 3.3. High Yields of Recombinant Adeno-Associated Virus Packaging

**Figure 3:**
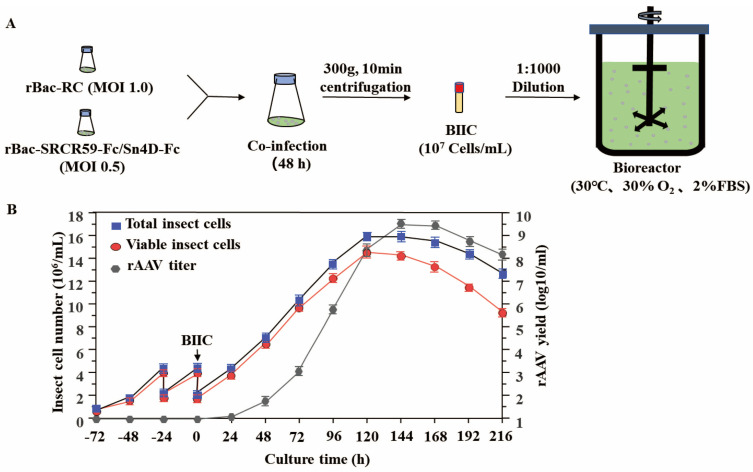
High yields of recombinant adeno-associated virus packaging. (**A**) The protocol of the rAAV-SRCR59-Fc/Sn4D-Fc large-scale production method. (**B**) Time course of rAAV production in the insect cell bioreactor. Sf9 cells (7.5 × 10^5^/mL) were grown in the 2 L bioreactor. Specifically, the cells were fed-batch with fresh medium twice, 48 h and 24 h before infection. BIICs were added to reach the final working volume when the cell density reached 2 × 10^6^ cells/mL (t = 0 hpi). Subsequently, the total insect cell density, viable insect cell density, and rAAV titer were measured every 24 h until 192 h post-infection.

The large-scale production protocol for rAAV-SRCR59-Fc/Sn4D-Fc is summarized in Figure 3A. The process was scaled up to 2 L insect cell rocking platform bioreactors based on the pilot study of optimization. Sf9 cells (7.5 × 10^5^/mL) were cultured in the bioreactor and subjected to fed-batch cultivation at t = −48 h and t = −24 h using fresh 2% FBS insect medium. BIICs were introduced into the final working volume when the cell density reached 2 × 10^6^ cells/mL (t = 0). The cells continued to proliferate, and the cell density increased until approximately t = 120 hpi. The highest titer of infectious viral particles (IVPs) of rAAV was achieved at t = 144 hpi (Figure 3B). The rAAV-SRCR59-Fc/Sn4D-Fc was harvested and purified from insect cell bioreactors. The initial yield of rAAV-SRCR59-Fc/Sn4D-Fc was 200 to 600 IVPs/cell and 5.37 ± 0.95 × 10^9^ IVPs/mL, with a recovery rate of 80.4 ± 2.9% and a purity of 83.1 ± 5.2%. In contrast, for the production of rAAV in cell flasks, the initial yield was 1–10 IVPs/cell and 3.19 ± 0.21 × 10^7^ IVPs/mL, with a recovery rate of 75.3 ± 3.1% and a purity of 89.7 ± 1.7%, respectively. As shown in Table 2, the comparison between the production in bioreactors and cell flasks clearly demonstrates the superiority of the large-scale production in terms of yield per cell and total IVPs/mL.

### 3.4. Identification of rAAV Produced and SVR Expressed In Vitro

**Figure 4:**
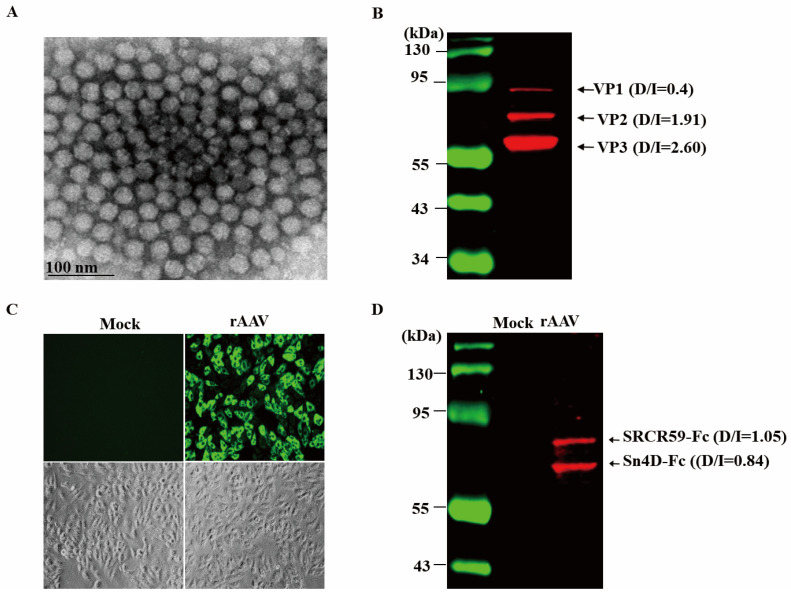
Identification of rAAV particles and SVR expression in rAAV-transduced 3D4/21 cells. (**A**) Purified rAAV-SRCR59-Fc/Sn4D-Fc was stained with 3% phosphotungstic acid and observed under a transmission electron microscope at an acceleration voltage of 75 kV. (**B**) The structural proteins of rAAV were detected by Western blotting using anti-AAV serum as the primary antibody. (**C**) 3D4/21 cells were transduced with rAAV-SRCR59-Fc/Sn4D-Fc and analyzed by immunofluorescence using anti-pig IgG. (**D**) The SVR fusions were purified from the cell medium of rAAV-transduced cells using Protein A affinity columns and analyzed by Western blotting using anti-Sn or anti-CD163 serum. D/I ratios represent target protein densitometry values normalized to loading control using Image J software 1.8.0. Western blot original images can be found in Supplementary Materials.

Electron microscopy demonstrated that the rAAV-SRCR59-Fc/Sn4D-Fc had the typical AAV morphology with a diameter of approximately 22 nm (Figure 4A). Western blotting confirmed that the rAAV reacted positively with an anti-AAV antibody with the standard VP1: VP2: VP3 ratio (Figure 4B). Immunofluorescent analysis demonstrated that 3D4/21 cells infected with rAAV-SRCR59-Fc/Sn4D-Fc were stained with FITC-labeled anti-pig IgG (Figure 4C). In addition to the identification of rAAV, the expression of SVR was also examined. Cell culture media were collected at 48 h, and the two SVR fusions were purified using Protein A Sefinose Columns. Western blotting analysis demonstrated two proteins with the expected molecular weights for SRCR59-Fc (88 kDa) and Sn4D-Fc (72 kDa) (Figure 4D).

### 3.5. Anti-PRRSV Activities of SVR Fusions

**Figure 5:**
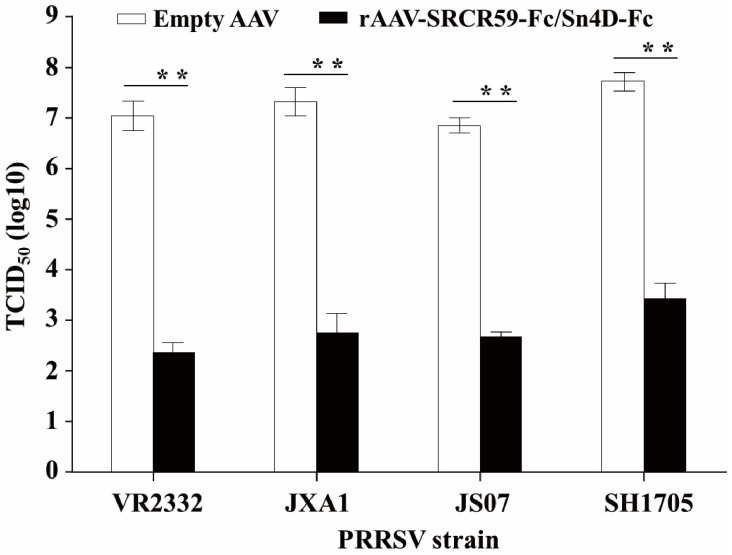
Antiviral activity of rAAV-SRCR59-Fc/Sn4D-Fc against diverse PRRSV-2 strains. 3D4/21 cells were transduced with rAAV-SRCR59-Fc/Sn4D-Fc (MOI 100) or empty AAV control. PAM cells were infected with PRRSV strains (MOI 0.5) and co-cultivated with transduced 3D4/21 cells for 24 h. Viral titers in PAM lysates were determined by TCID_50_ assay on Marc-145 cells. ** indicates highly significant difference (*p* < 0.01) between the empty AAV control and rAAV-SRCR59-Fc/Sn4D-Fc transduction experimental group. Error bars represent SD (*n* = 3).

The antiviral efficacy of dual soluble viral receptors (SVRs) was evaluated using a Transwell co-culture system (CORNING, Corning, NY, USA) to simulate viral inhibition in primary porcine alveolar macrophages (PAMs). Compared with the empty AAV control group (VR2332: 7.0 ± 0.2 log_10_ TCID_50_/mL; JXA1: 7.2 ± 0.2 log_10_ TCID_50_/mL; JS07: 6.9 ± 0.1 log_10_ TCID_50_/mL; SH1705: 7.6 ± 0.2 log_10_ TCID_50_/mL), transduction with rAAV-SRCR59-Fc/Sn4D-Fc significantly reduced viral titers across all tested PRRSV-2 strains (Figure 5). Post-treatment titers were VR2332: 2.4 ± 0.2 log_10_ TCID_50_/mL (4.6 log reduction), JXA1: 2.8 ± 0.4 log_10_ TCID_50_/mL (4.4 log reduction), JS07: 2.6 ± 0.1 log_10_ TCID_50_/mL (4.3 log reduction), and SH1705: 3.4 ± 0.3 log_10_ TCID_50_/mL (4.2 log reduction). On average, viral titers were reduced by 4.3 ± 0.2 log_10_ TCID_50_/mL (*p* < 0.01, two-tailed Student’s *t*-test). A Kruskal–Wallis test confirmed no significant differences in antiviral responses among PRRSV-2 strains (*p* = 0.12), indicating broad-spectrum activity of the dual SVRs against genetically diverse isolates.

## 4. Discussion

The rAAV production system is predicated on the co-infection of two baculoviruses. The co-infection ratio of these two baculoviruses significantly impacts rAAV production. It has been demonstrated that altering the ratios of baculoviruses in the triplicate virtual co-infection system altered the yields of infectious rAAV [30]. Our study found that an MOI ratio of 0.5:1.0 for the two baculoviruses can achieve the highest rAAV yields. This optimal ratio might be associated with a balanced supply of viral components necessary for rAAV assembly. When the ratio exceeds 1.0:1.0, insect cells undergo rapid apoptosis, which undermines rAAV packaging. This could be due to an over-burden of viral genetic material or proteins, triggering cellular stress responses and ultimately leading to cell death. Cell density is a crucial factor influencing rAAV production. Owing to oxygen limitations and challenges in nutrient uptake at high cell densities, optimal cell productivity can be achieved by initiating the infection when the cell density reaches 1–2 × 10^6^ cells/mL in fresh medium [31]. In our research, we also observed this phenomenon. The highest rAAV yields were obtained from cell flasks with an initial insect cell density of 2.0 × 10^6^ cells/mL, as opposed to 3.0 × 10^6^ cells/mL. At higher cell densities, the competition for oxygen and nutrients intensifies, which may limit the metabolic activity of the cells and consequently reduce rAAV production. Therefore, maintaining an appropriate cell density is essential for efficient rAAV production. The concentration of fetal bovine serum (FBS) in the insect growth medium also plays a role. A high FBS concentration elevates production costs. Moreover, excess nutrients lead to metabolite accumulation that inhibits cell growth. Based on these considerations, the optimal FBS concentration was determined to be 2%. This concentration strikes a balance between providing sufficient nutrients for cell growth and minimizing the negative effects of metabolite accumulation. It also helps in reducing the overall production cost, which is an important aspect for large-scale rAAV production. Temperature is another significant variable. Generally, insect cells are cultured at either 27 °C or 28 °C. Raising the culture temperature to 30 °C enhanced encapsidation, as determined by the ratio of capsids to DNase-resistant particles to infectious particles [32]. In our observations, there was no significant difference in Sf9 cell viability or baculovirus infection efficiency between 27 °C and 30 °C. Nevertheless, rAAV yields at 30 °C were indeed three times higher than those at 27 °C. The increased temperature may accelerate certain enzymatic reactions involved in rAAV assembly, such as the processes of capsid formation and genome encapsidation. However, further studies are needed to fully elucidate the underlying molecular mechanisms. In contrast to prior investigations, our study conducted a comprehensive assessment of multiple factors influencing rAAV production and precisely identified their optimal conditions. These findings not only offer invaluable perspectives for enhancing the efficiency of rAAV production but also establish a solid foundation for the large-scale manufacturing of rAAV.

Studies have indicated that a clinical dose for humans requires 10^12^–10^13^ rAAV vector particles, depending on the needed therapeutic protein expression level for treatment [33]. Considering the recombinant adeno-associated virus serotype 2 reference standard and the fact that the average human weighs 5–10 times more than a 20-day-old pig, at least 10^9^ infectious viral particles per piglet are necessary [34]. In this study, two baculoviruses (rBac-SRCR59-Fc/Sn4D-Fc and rBac-RC) co-infected Sf9 cells in cell flasks under optimized conditions. Unfortunately, the highest yield of rAAV-SRCR59-Fc/Sn4D-Fc was only about 2 × 10^7^ IVPS/mL at 72 h post-infection. This low titer from cell flasks is insufficient for experimental animal models and commercial production. Previous research has demonstrated that the baculovirus–insect cell (BIIC) system and fed-batch strategies are effective for rAAV packaging when three baculoviruses (rBac-GFP, rBac-Rep, or rBac-VP) co-infect insect cells [25,35]. Therefore, we employed a two-baculovirus co-infection system for rAAV-SRCR59-Fc/Sn4D-Fc production. First, we determined the optimal collection time (48 hpi) to optimize the BIIC process. Based on small- and mid-scale pilot studies, we scaled up the process to a 2 L stirred-tank single-use cell bioreactor with temperature and oxygen regulation. We controlled the Sf9 cell density and vitality through a fed-batch approach using a 2% FBS insect growth medium. As a result, the titer yield of rAAV-SRCR59-Fc/Sn4D-Fc in the bioreactor was significantly higher than that in small cell flasks, reaching 5.4 ± 0.9 × 10^9^ IVPS/cell, which is sufficient for commercial production and clinical gene therapy in piglets.

Soluble receptors derived from evolutionarily conserved host proteins represent a promising antiviral strategy, particularly for controlling rapidly mutating viruses with diverse serotypes [36,37]. This approach circumvents the need for strain-specific vaccines and addresses the antigenic diversity of PRRSV-2, as demonstrated by our in vitro neutralization assays showing comparable activity of rAAV-delivered Sn4D-Fc and SRCR59-Fc against genetically divergent PRRSV strains (Figure 5). Notably, while murine models validated the sustained SVR expression (35 days) enabled by rAAV vectors, direct translation to porcine hosts warrants caution due to species-specific immune responses [15]. In contrast to rAd vectors, which induced transient SVR persistence (<14 days) in pigs, the enhanced durability of rAAV-SVRs arises from the vector’s low immunogenicity and the use of porcine-derived SVR sequences, which minimize neutralizing antibody formation—a critical advantage for repeated veterinary administrations [14]. However, several limitations must be acknowledged. First, in vitro neutralization does not fully recapitulate the complexity of viral pathogenesis in vivo. Second, the long-term safety of rAAV-SVRs in pigs remains uncharacterized, including potential off-target effects and tissue tropism. Future studies should prioritize in vivo challenge experiments, dose optimization, and combination therapies with existing vaccines to maximize protective efficacy. Given the challenges in developing novel PRRS vaccines and the broad-spectrum neutralization capacity of these SVRs, rAAV vectors may offer an efficient alternative for PRRS control. 

## 5. Conclusions

This study establishes an optimized insect cell–baculovirus system for scalable production of rAAV vectors expressing dual soluble viral receptors (SVRs) against PRRSV. Through parameter optimization, high-titer rAAV-SRCR59-Fc/Sn4D-Fc (5.4  ±  0.9  ×  10^9^ IVPS/mL) was achieved in a 2 L bioreactor. In vitro assays confirmed efficient SVR expression and broad-spectrum antiviral activity, reducing viral titers of diverse PRRSV strains by ~4.3 log. This platform offers a viable strategy for large-scale rAAV manufacturing, highlighting the potential of rAAV-delivered SVRs as a durable, pan-strain therapeutic to address PRRSV challenges in swine production.

## Figures and Tables

**Table 1:** The PCR primers used in this study.

Gene	Primer Pair	Sequence (5′→3′)	Amplicon (bp)	Reference
AAV-Rep (of rBac-RC)	Sense	TGGTGGACGAGTGCTACA	113	[15]
	Antisense	CTCCGTGAGATTCAAACAGG		
P_CMV_ (of Bac-SRCR59-Fc/Sn4D-Fc)	Sense	ATGGTGATGCGGTTTTGGC	123	[15]
	Antisense	AGTCCCGTTGATTTTGGTG		

**Table 2:** A comparison of production and purification performance is conducted between different rAAV packaging strategies.

Packaging Strategy	Yield (IVPs/mL)	Yield (IVPs/Cell)	Recovery (%)	Purity (%)
Cell flask (72 h)	1.9 ± 0.3 × 10^7^	1~10	75.3 ± 3.1	89.7 ± 1.7
Cell bioreactor (144 h)	5.4 ± 0.9 × 10^9^	200~600	80.4 ± 2.9	83.1 ± 5.2

The purification results were determined by analyzing the protein grey value using Image Lab™ software 5.2.1.

## Data Availability

All data generated or analyzed during this study are included in this published article.

## Supplementary Materials

The following supporting information can be downloaded at: https://www.mdpi.com/article/10.3390/vetsci12040366/s1, File S1: Western blot original images.
