# Supplementary figures and images for: Scalable Production of Recombinant Adeno-Associated Virus Vectors Expressing Soluble Viral Receptors for Broad-Spectrum Inhibition of Porcine Reproductive and Respiratory Syndrome Virus Type 2
Source: Vet Sci. 2025 Apr 14;12(4):366. doi: 10.3390/vetsci12040366 (PMC12031001; doi:10.3390/vetsci12040366)

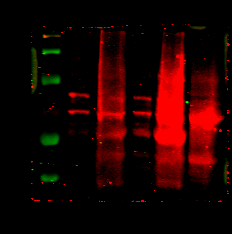

Supplement: Supplementary file 1 [file vetsci-12-00366-s001.zip › Fig.4DS.tiff]
